# A SAR Target Recognition Method via Combination of Multilevel Deep Features

**DOI:** 10.1155/2021/2392642

**Published:** 2021-11-26

**Authors:** Junhua Wang, Yuan Jiang

**Affiliations:** ^1^Institute of Engineering, Guangzhou College of Technology and Business, Gangzhou 510850, China; ^2^Ideological and Political Theory Teaching Department, South China Business College Guangdong University of Foreign Studies, Gangzhou 510545, China

## Abstract

For the problem of synthetic aperture radar (SAR) image target recognition, a method via combination of multilevel deep features is proposed. The residual network (ResNet) is used to learn the multilevel deep features of SAR images. Based on the similarity measure, the multilevel deep features are clustered and several feature sets are obtained. Then, each feature set is characterized and classified by the joint sparse representation (JSR), and the corresponding output result is obtained. Finally, the results of different feature sets are combined using the weighted fusion to obtain the target recognition results. The proposed method in this paper can effectively combine the advantages of ResNet and JSR in feature extraction and classification and improve the overall recognition performance. Experiments and analysis are carried out on the MSTAR dataset with rich samples. The results show that the proposed method can achieve superior performance for 10 types of target samples under the standard operating condition (SOC), noise interference, and occlusion conditions, which verifies its effectiveness.

## 1. Introduction

By processing high-resolution images obtained by synthetic aperture radar (SAR), analysis and interpretation of focus areas or targets of interest can be achieved. SAR target recognition technology can be used for reconnaissance and intelligence interpretation [1–3]. Since the 1990s, the SAR target recognition method has been enriched and progressed with the development of pattern recognition and artificial intelligence technology and has made considerable progress. Mainstream SAR target recognition methods usually use a two-stage process of feature extraction and classification to confirm the target label of unknown samples. Typical target features of SAR images include geometric shapes [4–7], projection transformations [8–12], and electromagnetic scattering [13–16]. Target contours, regions, shadows, etc., are representative shape features, which have the ability to distinguish different categories. Projection transformation algorithms include mathematical projection and transformation domain decomposition. The former includes matrix decomposition and manifold learning, and the latter includes wavelet, monogenic signal, and mode decomposition. The electromagnetic scattering characteristics reflect the backscattering characteristics of the target, such as peak value, scattering center, and polarization. The classification stage is closely coupled with feature extraction, and the difference of features is used to determine the category of the input sample. Nearest neighbor classifiers [17–19], support vector machine (SVM) [20–24], and sparse representation-based classification (SRC) [25–30] are the most widely used classifiers in existing SAR target recognition methods. With the rapid development of deep learning technology in recent years, deep learning models represented by convolutional neural network (CNN) [31–38] have been also employed in SAR target recognition.

Based on the existing research studies, this paper proposes a SAR target recognition method combining multilevel deep features. In the feature learning stage, the deep residual network (ResNet) [39–43] is used to learn the target multilevel feature maps. Compared with traditional handcrafted features, the feature maps obtained from ResNet have the advantage of stronger descriptive ability and can provide more sufficient discriminative information for the decision-making stage [44, 45]. Considering the possible correlation between multilevel depth features, this paper uses vector correlation as the basic criterion to perform cluster analysis on different deep features to obtain multiple depth feature sets. Afterwards, the joint sparse representation (JSr) is used to characterize and classify different feature sets, so as to further utilize their internal relations. Finally, the results of different feature sets are linearly weighted and fused to obtain reliable recognition results. In the experiment, the standard operating condition (SOC) and typical extended operating conditions (EOC) are set based on the MSTAR dataset to test and verify the method, and the results show its effectiveness and robustness.

## 2. Learning of Deep Features by ResNet

ResNet was proposed by Kaiming He and has been fully verified in a number of image detection and segmentation competitions [20, 21]. With the continuous increase of the number of network layers, the learned features become more abundant, which can better reflect the multifaceted characteristics of the target of interest in the image. However, at the same time, it will also lead to a serious gradient disappearance problem. For this reason, ResNet proposes residual learning to overcome the difficulty of network optimization. Assuming that *H*(*x*) represents the best mapping, the stacked nonlinear layer is used to obtain a new mapping *F*(*x*)=*H*(*x*) − *x*, and then, the current best mapping *F*(*x*)=*H*(*x*)+*x* is obtained. *F*(*x*)+*x* can be obtained by adding a “quick connection” operation in the feedforward network. This operation has the advantages of high efficiency and robustness and will not bring additional computational complexity.

Existing research results have verified the effectiveness of ResNet in the field of image processing (such as target detection and recognition). For this reason, this paper introduces it into SAR target recognition, which is mainly used for the learning and acquisition of the multilevel deep features. The ResNet structure used in this paper contains 20 layers in total. Compared with the general CNN, ResNet can realize the direct connection between the input and the subsequent nonadjacent layers, thereby minimizing the problems of information loss. ResNet simplifies the difficulty of network learning and improves overall training efficiency. The designed networks can learn multilevel feature maps of SAR images with rich descriptions. These features can reflect various characteristics of the target in the image from different aspects and can provide effective discriminative information for target recognition.

## 3. Clustering of Deep Features Based on the Correlation Principle

For the deep features acquired from the same SAR image, there may be some locality in their intrinsic correlation. For this reason, it is necessary to carry out correlation analysis on the multilevel deep features. This paper uses the traditional vector correlation as the criterion to design a deep feature clustering algorithm. Assuming that the multilevel deep feature obtained through ResNet is **V**={**I**_1_, **I**_2_,…, **I**_*N*_}, the correlation between every two different feature vectors is firstly calculated and recorded in Table 1. The subsequent Algorithm 1 is described,

In the above steps, the symbol “\” means the remainder operation; *c*_1**S**_*t*__ ≥ *T*_*c*_ indicates that the correlation coefficient **I**_1_ of each feature in **S**_*t*_ and is higher than the threshold *T*_*c*_. Generally, some empirical analysis and tests can be used to select a proper threshold. Under the condition of normalized similarity, the threshold value generally tends to the middle value of the interval to ensure the balance of feature correlation and independence. After the above clustering algorithm, the original *N* feature vectors are redivided into several feature sets. For a subset containing multiple feature vectors, they share relatively high internal correlation.

## 4. Recognition Method via Combination of Multilevel Deep Features

### 4.1. Principle of JSR

JSR is a multitask learning algorithm, mainly for multiple related sparse representation problems [10–13]. For the multiple deep feature vectors in the same feature set, this paper adopts JSR for characterization and classification. Let *M* feature vectors be **y**=[**y**^(1)^, **y**^(2)^,…, **y**^(*M*)^]; their independent sparse representation problem is as follows:

(1) $$\begin{array}{c} y^{\left(k\right)}=D^{\left(k\right)}\alpha^{\left(k\right)}+\varepsilon^{\left(k\right)},\;k=1,2,\ldots ,M, \end{array}$$

where **D**^(*k*)^, **α**^(*k*)^, and *ε*^(*k*)^ correspond to the dictionary sparse coefficient vector and representation error of the *k*th feature, respectively.

The problem of sparse representation of the *M* features can be jointly investigated, and the model is obtained as follows:

(2) $$\begin{array}{c} \underset{\beta }{\min}\left\{g\left(\beta \right)=\sum_{k=1}^{M}\left\|y^{\left(k\right)}-D^{\left(k\right)}\alpha^{\left(k\right)}\right\|\right\}, \end{array}$$

where **β**=[**α**^(1)^, **α**^(2)^,…, **α**^(*M*)^] is the matrix containing all the sparse coefficient vectors.

The joint representation model shown in formula (2) is only unified in form, but does not use the correlation between different features. The JSR model improves the overall solution accuracy by appropriately constraining the sparse matrix **β**, which is expressed as follows:

(3) $$\begin{array}{c} \begin{array}{c} \underset{\beta }{\min}\;g\left(\beta \right)+\lambda {\left\|\beta \right\|}_{2,1} \end{array}, \end{array}$$

where ‖·‖_2,1_ is the *ℓ*_1_/*ℓ*_2_ norm. According to the sparse coefficient matrix obtained by formula (3), the reconstruction errors of different categories can be calculated, respectively, and then, the decision of the target category can be generated as follows:

(4) $$\begin{array}{c} \text{identity}\left(y\right)=\underset{i}{\min}\sum_{k=1}^{M}\left\|y^{\left(k\right)}-D_{i}^{\left(k\right)}\alpha_{i}^{\left(k\right)}\right\|. \end{array}$$

### 4.2. Target Recognition via Decision Fusion

This paper uses multilevel deep feature clustering to effectively investigate the independence and relevance of these features. Then, the JSR is used to independently analyze each feature set with inherent correlation to obtain the reconstruction errors. Denote the output reconstruction error of each feature set as *f*_*t*_(*i*), *t*=1,2,…, *P*, and the linear weighting is employed to fuse them as follows:

(5) $$\begin{array}{c} e\left(i\right)=\omega_{1}f_{1}\left(i\right)+\omega_{2}f_{2}\left(i\right)+\cdots +\omega_{P}f_{P}\left(i\right), \end{array}$$

where *ω*_*i*_(*i*=1,2,…, *P*) denotes the weight coefficient.

This paper determines the weights according to the number of features in each feature set and sets *ω*_*i*_=*p*_*i*_/*P*, where *p*_*i*_ is the number of features in the *i*th feature set. Finally, the target category is determined according to the weighted reconstruction error of each category.

**Figure 1:**
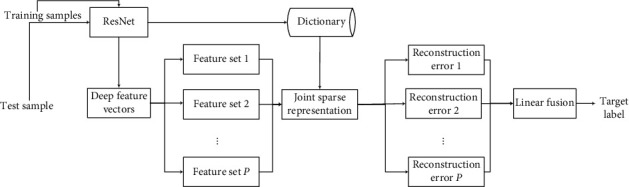
Flowchart of the proposed method.

Figure 1 shows the basic flow of the method in this paper with several main steps, including the deep feature clustering, JSR, and decision fusion. The final recognition performance is improved by examining the independence and correlation of multilevel deep features.

## 5. Experiments and Analysis

### 5.1. MSTAR Dataset

**Figure 2:**
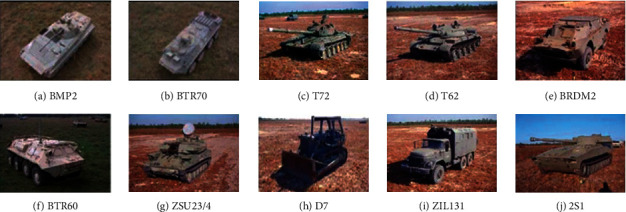
Images of targets to be classified. (a) BMP2. (b) BTR70. (c) T72. (d) T62. (e) BRDM2. (f) BTR60. (g) ZSU23/4. (h) D7. (i) ZIL131. (j) 2S1.

The MSTAR dataset is used to carry out experiments to test and analyze the performance of the method. The dataset contains 10 types of targets shown in Figure 2, and the related information of these SAR images is listed in Table 2.  Table 3 sets the training and test sets used in the experiments, including the categories, configurations, number of samples, and depression angles of 10 types of targets.

In the experiments, the focus is on the comparative analysis of the proposed method and existing four types of SAR target recognition methods, which are, respectively, denoted as “ResNet,” “A-ConvNet,” “JSR-Mono,” and “JSR-Deep.” Among them, both ResNet and A-ConvNet are methods based on deep learning models, using specific network structures for SAR target recognition. JSR-Mono and JSR-Deep use JSR as the classifier, but the difference is that the features used are monogenic signal and deep features.

### 5.2. Results and Analysis

#### 5.2.1. SOC

**Figure 3:**
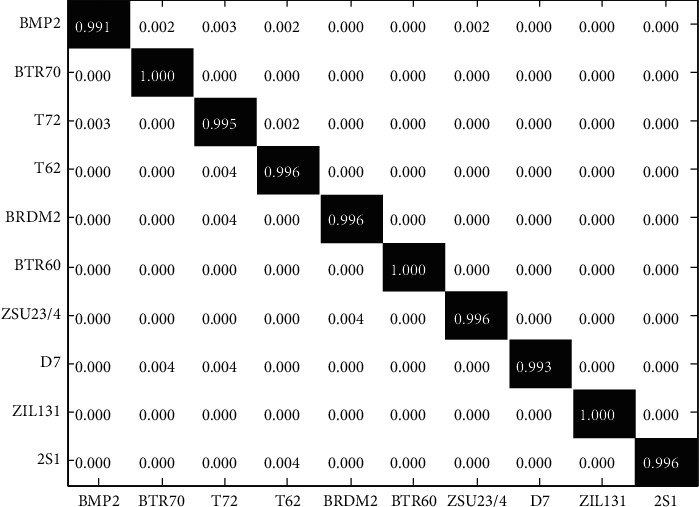
Confusion matrix achieved by the proposed method.

According to the settings in Table 3, the original samples in the MSTAR dataset are used for the validation. At this time, the experimental scene can be considered as a SOC, that is, the overall similarity between the test and training samples is relatively high. In the current experiment, the relevant threshold is set to 0.4.  Figure 3 shows the recognition results of the proposed method. The diagonal elements in the confusion matrix are the correct recognition rates of the corresponding target. It can be seen from Table 3 that the test configurations of BMP2 and T72 are more than the training ones, which leads to their relatively low recognition rate among the 10 types of targets. Synthesizing the results of 10 types of target recognition, Table 4 compares the average recognition rates of different methods in the current scenario. In terms of recognition accuracy, the method in this paper has better performance under current conditions, reflecting its effectiveness. Compared with the ResNet method, this paper further improves the recognition performance through the comprehensive application of the multilevel deep features. Compared with the JSR-Deep method, this paper promotes the improvement of the final recognition performance by introducing the screening analysis of deep features and the feature set decision and fusion.

According to the feature clustering algorithm, the threshold has an important influence on the final clustering result. Therefore, it is very important to select an appropriate clustering threshold.  Table 5 shows the average recognition rate of the proposed method at different thresholds, which achieves the best effect at the one of 0.4. If the threshold is too small, the constraint on the correlation between different features is too weak, that is, the features with large differences are clustered into one category. On the contrary, when the threshold is too large, the constraint on the correlation between different features is too strong. Individual features tend to call themselves one category, losing the value of cluster analysis. According to this result, this paper determines the cluster correlation threshold as 0.4 in the subsequent experiments.

#### 5.2.2. Noise Interference

Whether it is an optical image or a radar image, it is inevitably contaminated by noise during the acquisition process. In practical recognition systems, training samples are often carefully selected and preprocessed and have high image quality and signal-to-noise ratio (SNR). However, the test samples come from relatively random acquisition conditions, and it may be with poor image quality and low SNR. For this reason, the noise robustness of the recognition algorithm is very important. In this experiment, on the basis of the training and test sets in Table 3, noises are added to the test samples of 10 types of targets to obtain multiple test sets with different SNRs [5]. Then, various methods are tested separately.  Table 6 shows the results of the recognition rate in the current experimental scenario. Compared with the results under SOC, the performance of various methods under noise interference has been degraded. Observing the results under each SNR, the method in this paper can achieve the highest average recognition rate at each noise level, reflecting its noise robustness.

According to [10–13], sparse representation has a certain robustness to noise interference, which is also reflected in the stronger noise robustness of the sparse representation method in Table 6. On the one hand, the method in this paper uses multilevel deep features to complement each other to improve the ability to adapt to noise. At the same time, the JSR is used in the classification process, and the noise robustness can be further enhanced.

#### 5.2.3. Partial Occlusion

**Figure 4:**
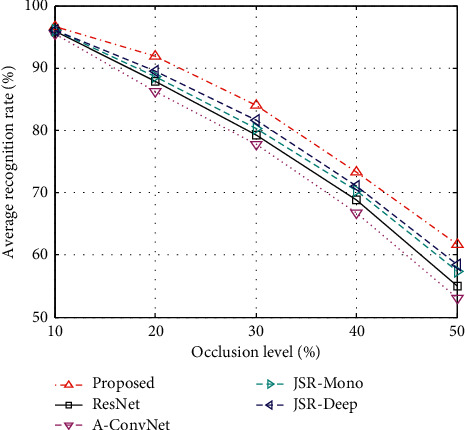
Average recognition rates under target occlusions.

Similar to the case of noise interference, the actual sample to be identified may also be partially occluded by the target. At this time, only part of the target characteristics can be reflected in the test sample and used for classification. According to the algorithm described in [5], on the basis of the test set in Table 3, the target area is partially occluded to obtain the test set under different occlusion ratios, and then, the performance of various methods is tested.  Figure 4 shows the recognition rate curve of each method. It can be seen that the method in this paper is more robust in this experimental scenario. Similar to the case of noise interference, the method based on JSR is more robust than the comparison methods. The proposed method in this paper combines the advantages of multilevel deep features, and JSR improves the overall performance of the recognition method under target occlusion conditions.

## 6. Conclusion

This paper proposes a SAR target recognition method combining multilevel deep features. This method first uses ResNet to learn SAR images to obtain multilevel deep feature vectors. Then, the deep feature vectors are clustered based on the correlation criterion to obtain multiple feature sets. On this basis, the different feature sets are characterized and classified based on JSR, and the reconstruction error results are obtained. Finally, the linear fusion analysis is performed on the results obtained from different feature sets to determine the target category. The proposed method can effectively combine the advantages of ResNet and JSR to improve recognition performance. Validation experiments are carried out on the MSTAR dataset, and the results show that the proposed method can achieve superior performance compared with existing methods under SOC and typical EOCs.

## Figures and Tables

**Algorithm 1:**
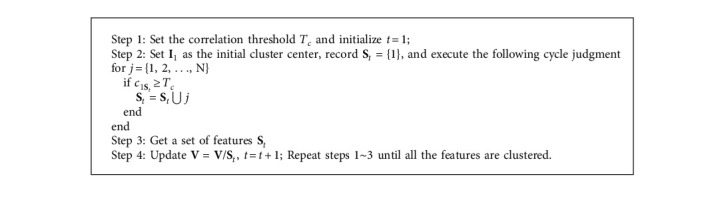
Clustering algorithm for deep features.

**Table 1:** Correlation matrix of deep feature vectors.

	**I**_1_	**I**_2_	⋯	**I**_*N*_
**I**_1_	*c*_11_	*c*_12_	⋯	*c*_2*N*_
**I**_2_	*c*_21_	*c*_22_	⋯	*c*_2*N*_
⋮	⋮	⋮	⋱	⋮
**I**_*N*_	*c*_*N*1_	*c*_*N*2_	⋯	*c*_*NN*_

**Table 2:** Relevant information about MSTAR dataset.

Azimuth (°)	Depression angle (°)	Resolution (m)	Size (pixel)
0∼360	15, 17, 30, 45	0.3 × 0.3	128 × 128

**Table 3:** Training and test sets for the 10-class recognition problem.

Class	Training		Test	
	Configuration	Samples	Configuration	Samples
BMP2	9563	233	9563	195
			9566	196
			C21	196

BTR70	C71	233	C71	196
			132	196

T72	132	232	812	195
			s7	191

T62	A51	299	A51	273
BRDM2	E-71	298	E-71	274
BTR60	7532	256	7532	195
ZSU23/4	d08	299	d08	274
D7	13015	299	13015	274
ZIL131	E12	299	E12	274
2S1	B01	299	B01	274

**Table 4:** Average recognition rates under the standard operating condition.

Method	Average recognition rate (%)
Proposed	99.28
ResNet	99.02
A-ConvNet	98.75
JSR-mono	98.68
JSR-deep	99.14

**Table 5:** Average recognition rates of the proposed method at different thresholds.

*T*_*c*_	0.1	0.2	0.3	0.35	0.4	0.45	0.5	0.55
Average recognition rate (%)	99.08	99.12	99.18	99.24	99.28	99.22	99.15	99.10

**Table 6:** Recognition rates under noise corruption.

Methods	SNR/dB				
	−10	−5	0	5	10
Proposed	70.58	81.32	88.14	93.56	98.94
ResNet	63.42	74.42	83.43	87.53	98.42
A-ConvNet	62.74	73.46	82.81	86.78	98.02
JSR-Mono	64.92	75.08	85.09	89.02	98.13
JSR-Deep	66.57	76.82	85.49	91.82	98.36

## Data Availability

The dataset used in this paper can be accessed upon request.

## References

[1] El-Darymli K., Gill E. W., Power D., Moloney C. (2016). Automatic target recognition in synthetic aperture radar imagery: a state-of-the-art review. *IEEE Access*.

[2] Amoon M., Rezai‐rad G. a. (2014). Automatic target recognition of synthetic aperture radar (SAR) images based on optimal selection of Zernike moments features. *IET Computer Vision*.

[3] Ding B., Wen G., Ma C., Yang X. (2016). Target recognition in synthetic aperture radar images using binary morphological operations. *Journal of Applied Remote Sensing*.

[4] Shan C., Huang B., Li M. (2018). Binary morphological filtering of dominant scattering area residues for SAR target recognition. *Computational Intelligence and Neuroscience*.

[5] Jin L., Chen J., Peng X. (2019). Synthetic aperture radar target classification via joint sparse representation of multi-level dominant scattering images. *Optik*.

[6] Tan J., Fan X., Wang S. (2019). Target recognition of SAR images by partially matching of target outlines. *Journal of Electromagnetic Waves and Applications*.

[7] Papson S., Narayanan R. M. (2012). Classification via the shadow region in SAR imagery. *IEEE Transactions on Aerospace and Electronic Systems*.

[8] Mishra A. K. Validation of PCA and LDA for SAR ATR.

[9] Mishra A. K., Motaung T. Application of linear and nonlinear PCA to SAR ATR.

[10] Cui Z., Cao Z., Yang J., Feng J., Ren H. (2015). Target recognition in synthetic aperture radar images via non‐negative matrix factorisation. *IET Radar, Sonar & Navigation*.

[11] Yu M., Dong G., Fan H., Kuang G. (2018). SAR target recognition via local sparse representation of multi-manifold regularized low-rank approximation. *Remote Sensing*.

[12] Huang Y., Peia J., Yanga J., Wang B., Liu X. (2014). Neighborhood geometric center scaling embedding for SAR ATR. *IEEE Transactions on Aerospace and Electronic Systems*.

[13] Xiong W., Cao L., Hao Z. Combining wavelet invariant moments and relevance vector machine for SAR target recognition.

[14] Dong G., Kuang G., Wang N., Zhao L., Lu J. (2015). SAR target recognition via joint sparse representation of monogenic signal. *IEEE Journal of Selected Topics in Applied Earth Observations and Remote Sensing*.

[15] Zhou Y., Chen Y., Gao R., Feng J., Zhao P., Wang Li (2019). SAR target recognition via joint sparse representation of monogenic components with 2D canonical correlation analysis. *IEEE Access*.

[16] Chang M., You X., Cao Z. (2019). Bidimensional empirical mode decomposition for SAR image feature extraction with application to target recognition. *IEEE Access*.

[17] Potter L. C., Moses R. L. (1997). Attributed scattering centers for SAR ATR. *IEEE Transactions on Image Processing*.

[18] Ding B., Wen G., Zhong J., Ma C., Yang X. (2017). A robust similarity measure for attributed scattering center sets with application to SAR ATR. *Neurocomputing*.

[19] Ding B., Wen G., Huang X., Ma C., Yang X. (2017). Target recognition in synthetic aperture radar images via matching of attributed scattering centers. *IEEE Journal of Selected Topics in Applied Earth Observations and Remote Sensing*.

[20] Zhao Q., Principe J. C. (2001). Support vector machines for SAR automatic target recognition. *IEEE Transactions on Aerospace and Electronic Systems*.

[21] Tison C., Pourthie N., Souyris J. Target recognition in SAR images with support vector machines (SVM).

[22] Demirhan M. E., Salor Ö. Classification of targets in SAR images using SVM and k-NN techniques.

[23] Liu H., Li S. (2013). Decision fusion of sparse representation and support vector machine for SAR image target recognition. *Neurocomputing*.

[24] Thiagaraianm J. J., Ramamurthy K. N., Knee P., Spanias A., Berisha V. Sparse representations for automatic target classification in SAR images.

[25] Song H., Ji K., Zhang Y., Xing X., Zou H. (2016). Sparse representation-based SAR image target classification on the 10-class MSTAR data set. *Applied Sciences*.

[26] Ding B., Wen G. (2018). Sparsity constraint nearest subspace classifier for target recognition of SAR images. *Journal of Visual Communication and Image Representation*.

[27] Li W., Yang J., Ma Y. (2020). Target recognition of synthetic aperture radar images based on two-phase sparse representation. *Journal of Sensors*.

[28] Yu L., Wang L., Xu Y. (2021). Combination of joint representation and adaptive weighting for multiple features with application to SAR target recognition. *Scientific Programming*.

[29] Zhu X. X., Tuia D., Mou L. (2017). Deep learning in remote sensing: a comprehensive review and list of resources. *IEEE Geoscience and Remote Sensing Magazine*.

[30] Kang M., Ji K., Leng X., Xing X., Zou H. (2017). Synthetic aperture radar target recognition with feature fusion based on a stacked autoencoder. *Sensors*.

[31] Morgan D. E. Deep convolutional neural networks for ATR from SAR imagery.

[32] Chen S., Wang H., Xu F., Jin Ya-Q. (2016). Target classification using the deep convolutional networks for SAR images. *IEEE Transactions on Geoscience and Remote Sensing*.

[33] Zhao J., Zhang Z., Yu W., Truong T.-K. (2018). A cascade coupled convolutional neural network guided visual attention method for ship detection from SAR images. *IEEE Access*.

[34] Min R., Lan H., Cao Z., Cui Z. (2019). A gradually distilled CNN for SAR target recognition. *IEEE Access*.

[35] Kechagias-Stamatis O., Aouf N. (2019). Fusing deep learning and sparse coding for SAR ATR. *IEEE Transactions on Aerospace and Electronic Systems*.

[36] Jiang C., Zhou Y. (2018). Hierarchical fusion of convolutional neural networks and attributed scattering centers for Robust SAR ATR. *Remote Sensing*.

[37] Dong G., Wang N., Kuang G., Zhang Y. (2014). Kernel linear representation: application to target recognition in synthetic aperture radar images. *Journal of Applied Remote Sensing*.

[38] Xin Y., Kuan L., Jiao L. SAR automatic target recognition based on classifiers fusion.

[39] Cui Z., Cao Z., Yang J., Feng J. (2013). A hierarchical propelled fusion strategy for SAR automatic target recognition. *EURASIP Journal on Wireless Communications and Networking*.

[40] Srinivas U., Monga V. Meta-classifiers for exploiting feature dependence in automatic target recognition.

[41] Huan R., Pan Y. (2011). Decision fusion strategies for SAR image target recognition. *IET Radar, Sonar & Navigation*.

[42] Chang C., Lin C. (2011). LIBSVM: a library for support vector machines. *ACM Transactions on Intelligent Systems and Technology*.

[43] He K., Zhang X., Ren S., Sun J. Deep residual learning for image recognition.

[44] Gao M., Song P., Wang F., Liu J., Mandelis A., Qi DaW. (2021). A novel deep convolutional neural network based on ResNet-18 and transfer learning for detection of wood knot defects. *Journal of Sensors*.

[45] Jing E., Zhang H., Li Z., Liu Y., Ji Z., Ganchev I. (2021). ECG heartbeat classification based on an improved ResNet-18 model. *Computational and Mathematical Methods in Medicine*.

